# Delegation of medication administration from registered nurses to non-registered support workers in community care settings: A systematic review with critical interpretive synthesis

**DOI:** 10.1016/j.ijnurstu.2021.104121

**Published:** 2022-02

**Authors:** Colin B. Shore, Jill Maben, Freda Mold, Kirsty Winkley, Angela Cook, Karen Stenner

**Affiliations:** a School of Health Sciences, Faculty of Health & Medical Sciences, University of Surrey, Kate Granger Building, Priestly Road, Surrey Research Park, Guildford, Surrey GU2 7YH, United Kingdom; b King's College London & Florence Nightingale Faculty of Nursing, Midwifery & Palliative Care, James Clerk Maxwell Building, Waterloo Road, London SE1 8WA, United Kingdom; c Head of Nursing and Quality, Shropshire Community Health NHS Trust, William Farr House, Mytton Oak, Road, Shrewsbury SY3 8XL, United Kingdom

**Keywords:** Barriers to delegation, Community nursing, Facilitators to delegation, Healthcare assistant, Medicine administration, Nurse-delegation, Nursing assistant, Registered nurse

## Abstract

**Introduction:**

Healthcare workforces are currently facing multiple challenges, including aging populations; increasing prevalence of long-term conditions; and shortfall of registered nurses. Employing non-registered support workers is common across many countries to expand service capacity of nursing teams. One task delegated to non-registered support workers is medication administration, which is considered a complex task, with associated risks. This is an important topic given the predicted global increase in patients requiring assistance with medication in community settings. This review explores the evidence on delegation of medication administration from registered nurse to non-registered support workers within community settings, to better understand factors that influence the process of delegation and its impact on service delivery and patient care.

**Methods:**

The review followed key principles of Critical Interpretative Synthesis and was structured around Preferred Reporting Items for Systematic Reviews and Meta‐analysis guidelines. Literature searches were conducted in MEDLINE, CINAHL, Embase, and ProQuest-British Nursing Index databases. Twenty studies were included.

**Results:**

Findings are reported under four themes: 1, Regulatory and contextual factors; 2, Individual and team level factors; 3, Outcomes of delegation; and 4, Process of implementation and evaluation. Delegation was found to be a complex phenomenon, influenced by a myriad of interconnecting factors at the *macro, meso, micro* level. At the macro level, the consistency and clarity of government and state level regulations was found to facilitate or impede delegation of medication administration. Lack of clarity at the macro level, impacted at *meso* and micro levels, resulting in confusion around what medication administration could be delegated and who held responsibility. At the micro level, central to the interpretation of success was the relationship between the delegator and delegatee. This relationship was influenced by personal views, educational and systems factors. Many benefits were reported as an outcome of delegation, including service efficiency and improved patient care. The implementation of delegating medication administration was influenced by regulatory factors, communication, stakeholder engagement, and service champions.

**Conclusion:**

Delegation of medication administration is a complex process influenced by many interrelating factors. Due to the increased risk associated with medication administration, clear and consistent regulatory and governance frameworks and procedures are crucial. Delegation of medication administration is more acceptable within a framework that adequately supports the process, backed by appropriate policy, skills, training, and supervisory arrangements. There is a need for further research around implementation, clinical outcomes and medication errors associated with delegation of medication administration.

**What is already known**

• The administration of medicines can be a complex and time-consuming procedure that requires in-depth knowledge of the medicine and its intended effect.
• Within acute care setting, clarity of roles, responsibilities, education, and effective inter-professional and team relationships have all been identified as important factors in registered nurse to non-registered support worker delegation.

**What this paper adds**

• Registered nurses in several countries delegate medicines administration to non-registered support workers in a range of community settings, in order to improve service efficiency.
• The success and safety of delegation is influenced by multiple factors and requires robust training and governance to build and support trusting relationships between delegator and delegatee.
• This review presents a framework that visually demonstrates the complex interactions that may influence successful delegation. This consolidated evidence base may act as a reference point for any community nursing team looking to implement delegation of medication administration.

## 1 Introduction

The global healthcare workforce is facing numerous challenges with increasing populations, longer life expectancies and increased burden of long-term conditions (Bates et al., 2016). A healthcare workforce that is of adequate size and skills, is critical to the attainment of any population health goal (WHO, 2016). However, many countries face difficulties in the training and retention of their healthcare workforce (WHO, 2016; Buchan et al., 2019). There is an estimated global healthcare workforce shortage of 7.2 million; predicted to grow to 12.9 million by 2030, including a shortfall of 5.9 million nurses (WHO, 2016, 2020a). In the United Kingdom (UK), as in other countries such as Australia and Italy (Shepperd et al., 2009), there is a desire to support healthcare provision in the community and prevent hospital admissions (RCN, 2014). With predicted shortfalls of 108,000 nurses in the UK by 2030, substantial investment in the community nursing workforce will be needed over the next 10 years (Buchan et al., 2019; Kings Fund, Closing the Gap, 2019; NHS Long Term Plan, 2019). This community nursing shortage has been exacerbated during the COVID-19 pandemic (WHO, 2020b).

The employment of non-registered healthcare workers (such as healthcare assistants, nursing assistants, assistant practitioners, medication technicians) is common across many countries to expand service capacity in nursing teams (Blay and Roche, 2020). In this review, the term ‘non-registered support worker’ is used to encompass these many different titles. In the UK, there has been an 11% increase in non-registered support workers between 2014 and 2018 (Buchan et al., 2019); comprising approximately 24% of the NHS healthcare workforce (*n* = 106,500) (The Cavendish Review, 2013), with approximately 16,968 employed in community health services (Spilsbury et al., 2013). However, this does not account for those working outside of the NHS, employed by local authorities or private organisations who might provide care for NHS patients. Similar increases in the non-registered support workers workforce have been reported in Australia and the United States of America (USA) (Blay and Roche, 2020). Non-registered support workers are part of the broader nursing team in acute, primary and community care settings. In community settings, this can include homecare, residential homes, assisted living facilities and nursing homes (Kessler et al., 2010; Bosley and Dale, 2008).

Non-registered support workers do not hold a qualification accredited by a professional association and are not formally regulated by a statutory body (Kessler et al., 2010). As such, it is common practice for registered nurses to delegate nursing tasks to non-registered support workers (Gillan and Graffin, 2010). In this review we adopt the Nursing and Midwifery Council (NMC) 2018 definition of delegation as ‘*the transfer to a competent individual, of the authority to perform a specific task in a specified situation’*. Importantly, the registered nurse retains legal responsibility for the delegated nursing care, as is stipulated in multiple international standards (ANA and NCSBN, 2019; Intercollegiate information paper developed by the CSP, RCSLT, BDA and the RCN, 2006). Clarity of roles and responsibilities (Munn et al., 2013; Blay and Roche, 2020), effective inter-professional and team relationships (12Campbell et al., 2020, Hopkins et al., 2012) and the quality of supervision ((Bifarin and Stonehouse, 2017)oe have all been identified as important factors in nurse to non-registered support worker delegation. There is mixed evidence that non-registered support workers provide care of equivalent standard to registered nurses (12Griffiths et al., 2019, Hopkins et al., 2012), reports of missed opportunities to provide care (Bittner and Gravlin, 2009; Kalisch, 2006) and potential errors/risks to patient care in different settings (Potter et al., 2010; Kalisch, 2011; RCN, 2017). While nurse to non-registered support worker delegation has been reviewed in acute care settings, a lack of evidence is reported for community settings (Blay and Roche, 2020; Munn et al., 2013; Hewko et al., 2015). It is therefore important to review delegation in the community context where non-registered support workers may undertake different types of activities with less opportunity for direct contact with registered nurses (Blay and Roche, 2020; Bosley and Dale, 2008) and where job stability may be lower than other settings (Hewko et al., 2015).

Non-registered support worker activity have been categorised as mainly direct care involving routine tasks associated with personal hygiene and mobilisation (Blay and Roche, 2020), however, some are delegated medication administration (Kesler, Spilsbury, Heron 2014; Dickens et al., 2008) and other tasks previously restricted to registered nurses, such as electrocardiograms, cannulation, and sutures (Blay and Roche, 2020; Spilsbury et al., 2013). The preparation and administration of medication are considered a complex higher-grade task (Blay and Roche, 2020), with associated risks to healthcare and costs (Assiri et al., 2018). As such, medication administration (the manner in which a medicine is administered) can be argued to require knowledge and competence to assess and administer safely (Blay and Roche, 2020). Prevalence of medication errors in community settings can be wide ranging and include errors of prescribing (5–94%), administering (44%) and monitoring (73%) (Assiri et al., 2018). The safe delegation of medication administration to non-registered support workers is an important topic given the predicted global increase in patients with long-term conditions who will require assistance with medication in community settings (Mangin et al., 2018; CQC, 2019). For these reasons, it is timely to review the research evidence on the delegation of medication administration from registered nurses to non-registered support workers within community settings.

### 1.1 Aim

The aim was to review evidence regarding the delegation of medication administration from registered nurse to non-registered support workers within community care settings to better understand factors that influence the process of delegation and its impact on service delivery and patient care.

## 2 Method

### 2.1 Study design and development

Critical Interpretative Synthesis (CIS) is an approach that adopts a systematic method for combining qualitative and quantitative research and an inductive approach to generating theory to further develop an understanding of a topic (Dixon-wood et al., 2006; Depraetere et al., 2020). This review followed key principles of Critical Interpretative Synthesis, with an emphasis on theory development, critical orientation, and flexibility (Dixon-Woods et al., 2006). However, flexibility can be a disadvantage if authors are not explicit about their review process, raising concerns about trustworthiness (Depraetere et al., 2020). To provide rigour and structure to the review processes, a pre-defined protocol was used in accordance with the Preferred Reporting Items for Systematic Reviews and Meta-Analyses statement (Moher et al., 2009) and registered with the International prospective register of systematic reviews (CRD42020201453).

Critical Interpretative Synthesis allows for the formulation of an open research question, which is then refined (Dixon-wood et al., 2006; Depraetere et al., 2020). The initial research questions were:

1. What are stakeholders’ views (registered nurses, non-registered support workers & patients) on the acceptance of and/or barriers and facilitators to the delegation of medication administration from registered nurse to non-registered support workers within community care settings?
2. What evidence is there of impact (service development, care delivery, medication errors, cost, and patient outcomes) associated with delegation of medication administration from registered nurses to non-registered support workers within community care settings?

During the process of data extraction and analysis (described below) the research question was extended to capture emergent factors and strategies associated with the implementation of medication administration delegation. Furthermore, it became apparent that data needed to be viewed and understood at the macro, *meso*, micro levels (described below) and a visual framework was created to present the complex nature of delegation (described below).

### 2.2 Search strategy and selection

A comprehensive, replicable search strategy was developed (CS, KS, and FM) to identify primary research on the delegation of medication administration from registered nurse to non-registered support workers in community settings. The search strategy was supported by the use of SPIDER (Sample, Phenomenon of Interest, Design, Evaluation, Research type) (Cooke et al., 2012). Staged purposive sampling, advocated for Critical Interpretative Synthesis (Dixon-wood et al., 2006; Depraetere et al., 2020) was performed, however, additional theoretical sampling was not necessary as the primary research evidence base was sufficient to answer the research questions.

Four international electronic databases were searched, starting with MEDLINE, and customised using index terms across three other databases (CINAHL, Embase, ProQuest-British Nursing Index), using a combination of free-text words within the title/abstract. Search terms related closely to the review questions, and included: ‘medication’, ‘delegation’, ‘community nursing’, ‘registered practitioner’ and ‘unlicensed practitioner’ (Appendix 1). Searches began from inception of electronic databases, 1949, up to July 2020, to ensure a wide scope of material. Alerts were set up for each database to ensure most current inclusion. Hand searches of Nursing Times, British Journal of Healthcare Assistants, International Journal of Nursing Studies, NHS Evidence website and grey literature sources (Social Science Research Network and Open Grey), were conducted until September 2020. Hand searches also included the review of reference lists of relevant papers and contact with known researchers working in this area. One thousand, five hundred and fifty-six search results were found across all databases.

The title of registered nurse is inclusive of titles that might be held in different countries (e.g., home health nurse, community nurse) registered according to their country's regulations. Multiple nomenclatures were used to describe non-registered support workers, including: Healthcare support worker; Healthcare assistants; Associate practitioners; Homecare aides; Medication aides; Non-registered practitioners; Unlicensed assistive personnel; Community care aides; and Medication technicians.

**Fig. 1:**
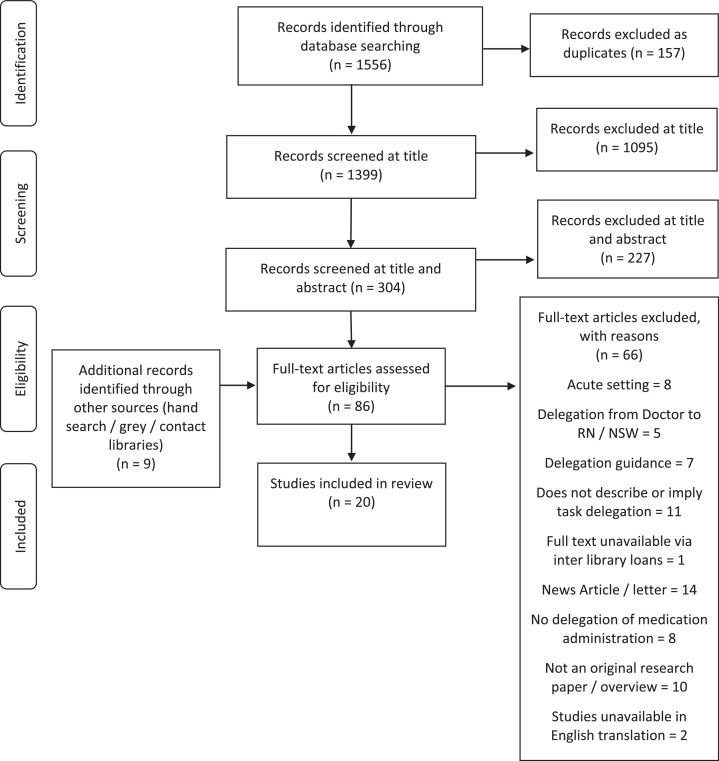
Preferred Reporting Items for Systematic Reviews and Meta-Analyses flow diagram depicting study selection, screening, eligibility for inclusion and analysis.

Once duplicates were excluded (*n* = 157), one member of the research team (CS) independently screened 1399 papers from their title. Three hundred and four papers were screened by title and abstracts against the inclusion and exclusion criteria by one author (CS), of which a subset of excluded papers was cross-referenced by another author (KS). Two authors (CS, KS), confirmed articles to be excluded at full text, discussing discrepancies until a consensus was reached. All studies satisfying the eligibility criteria (Table 1) were included (*n* = 20). Fig. 1 depicts the search process and the justification of excluding articles at multiple stages of the selection process.

**Table 1:** Inclusion and exclusion criteria used in collating evidence on delegation of medication administration from registered nurse to non-registered support workers in community care settings.

Language	English language unless translated versions were available. Any country of origin
Study	Any empirical study design, including service evaluation, that describes delegation of medication administration from an registered nurse to a non-registered support worker. Manuscripts that were commentary, opinion/editorial pieces, policy, or delegation guidance were excluded
Setting	Delegation must be within a community healthcare setting where a nurse delegates medication administration to an non-registered support workers
Population	Adults ages 18 years who are employed as registered nurse or non-registered support worker or where alternative job titles are used for example, Unlicensed Practitioner or Healthcare Assistant
Outcomes	Barriers to and/or facilitators of the delegation of medication administration; consumers’, carers’ and/or healthcare professionals’ perceptions and experiences of medication administration delegation Impact of delegation of medication administration; economic comparisons/patient, staff and/or educational satisfaction/expectation/service development/care delivery/medication errors and patient outcomes

### 2.3 Assessment of study quality

In line with Critical Interpretative Synthesis (Dixon-wood et al., 2006), a formal appraisal of methodological quality of individual papers was conducted; no studies were excluded on basis of quality. Comprehensiveness of reporting and transparency was evaluated using the Quality Assessment Tool for Studies with Diverse Designs (Sirriyeh et al., 2012); a tool demonstrated to produce good validity and test-retest reliability across a diversity of study designs (Fenton et al., 2015; Noblet et al., 2017). Two researchers (CS, KS) independently assessed and scored each study against the tool criteria and graded manuscripts against the tools 16 questions focusing on the methodology and design, for example, on evidence of sample size consideration, and rationale for choice of data collection tools. The Quality Assessment Tool for Studies with Diverse Designs is based on a 4-point scoring scale per question. Scores were combined, resulting in a mean (min-max) score for each paper (Sirriyeh et al., 2012) (Table 2). Independent scores were compared, with disagreements discussed (CS, KS) and resolved. Mean quality scores was 25 (7–39) out of a total possible score of 48. Appendix 2 and 3 provides a detailed breakdown of questions and independent grading of study manuscripts.

**Table 2:** Characteristics and quality of included studies (*n* = 20).

Paper ID	Lead author and year	Country	Setting	Study aims and methods	Study participants	Type of medication delegated	QATSDD(0 to 48)
A	(Axelsson and Elmstahl, 2004)	Sweden	Community home care	Assessment of homecare aide's engagement with medication administration and knowledge Quantitative survey	393 Home care aides	Knowledge about common drugs indications (β-blockers, Diuretics, Glyceryl trinitrate Acetyl salicylate, Paracetamol, Oxazepam, Zolpidem). Does not indicate if non-registered support workers were delegated these	29
B	Budden 2012	USA	Assisted living facilities, Nursing home, other long-term care (i.e., Community home care, other (i.e., Hospice)	Assessment of medication aides work setting, education, supervision, and role Quantitative survey	3455 Medication aides	**Injectables.** 29% (1002/3455) indicated they were allowed to administer medications by injection. Insulin only medication mentioned **Topicals.** 94% (3248/3455) of aides were allowed to administer topical medications. **Orals**. 82% (2833/3455 aides were allowed to administer sublingual medications and maintenance doses of oral anticoagulants **Tubes.** 20% (691/3455) allowed to administer medication via tubes **Classes of Drugs.** 90% (3109/3455) were allowed to administer controlled substances. 27% (933/3455) were allowed to administer chemotherapeutic agents (oral tablets tamoxifen)	28
C	Bystedt 2011	Sweden	Community home care	Describe perception of registered nurses on delegation to non-registered support workers Qualitative interviews	12 Registered nurses	Insulin mentioned as an example of what they could administer. No other medication indicated	34
D	Cook 2015	UK	Community home care	Describe the implementation of insulin delegation Mixed Methods in-house service evaluation	Since 2009, 565 non-registered practitioners have attended module 1, 490 have attended module 2 and 342 have attended module 3.	Insulin	7
E	Craftman 2012	Sweden	Community home care	Describe nurses’ perceptions of delegating administration of medication to non-registered support workers in the municipal social services Qualitative interviews	20 District nurses	Did not provide details of the types of medicines delegated. Described that delegation of medicines occurs in conjunction with other tasks	30
F	De Vliegher 2016	Belgium	Community home care	Explore experiences of registered nurses, non-registered support workers and managers on delegation of nursing activities, supervision and of delegation impact Qualitative interviews	12 Home nurses, 12 Health care assistants and 8 managers	Did not provide details of the types of medicines delegated. Described that delegation of medicines occurs in conjunction with other tasks	28
G	(Dupler et al., 2015)	USA	Nursing homes	Explore the beliefs and understanding of skilled nursing facility staff regarding the Washington State Medication Assistant Endorsement Program Quantitative cross-sectional survey	109 Nursing assistants trained as Medication assistants, 51 Registered nurses. 19 Licensed practical nurses, 3 and Nursing home administrators	Oral, topical, inhalation (oxygen) administration, schedule IV/V drugs	29
H	Dutton 2018	UK	Community home care	Describe the implementation of insulin delegation Mixed Methods in-house service evaluation	4 patients, 2 Healthcare support workers, 2 Community nurses	Insulin	7
I	(Gransjon-Craftman et al., 2016)	Sweden	Residential care homes	Describe nurses’ perceptions of delegating administration of medication to non-registered support workers in residential care homes Qualitative interviews	18 Registered nurses	Did not provide details of the types of medicines delegated. Described that delegation of medicines occurs in conjunction with other tasks	31
J	(Gransjon-Craftman et al., 2014)	Sweden	Community home care	Explore how non-registered support workers perceive administration of medication to older people living at home Qualitative interviews	19 home-care assistants	Did not provide details of the types of medicines delegated. Described that delegation of medicines occurs in conjunction with other tasks	24
K	Gregory 2019	UK	Community home care	Describe the implementation of insulin delegation Mixed Methods in-house service evaluation	6 healthcare assistants / associate practitioners	Insulin	7
L	Hughes 2006	USA	Nursing homes	To determine the relation between organizational characteristics and medication technician use and quantify the impact of medication technicians use of medications Quantitative cross-sectional survey	6344 Medicare/Medicaid certified nursing homes in 23 states	Antiosteoporosis medication was as reported in a case study. Further details were provided on state regulations on what can or cannot be delegated for administration Kentucky - CAN NOT: Convert drug dosages; administer injectable medications via tubes inserted into any body cavity; administer antineoplastic drugs Maine – CAN administer selected non injectable medications Missouri - CAN administer noninjectable medication and insulin (appears contradictory) Oklahoma - CAN give regularly scheduled, noninjectable medications. Oregon – CAN give (1) oral, sublingual, and buccal medications, including regularly scheduled controlled substances (narcotics); (2) eye medications; (3) ear, nose, rectal, and vaginal medications; (4) skin ointments, and topical medications including patches and transdermal medications; (5) medications by gastrostomy or jejunostomy; and (6) dosage-controlled aerosol/nebulizer therapies. They may also give “PRN” (as needed) medications as directed by RN(registered nurse)	32
M	Kapborg 1999	Sweden	Community home care	Analyse cases of malpractice concerning nurses within the municipal health and medical care Mixed Methods Interviews and cross-sectional assessment of records	8 Nurses, assessment of medical records of medical records over a 3-year period	Did not provide details of the types of medicines delegated. Described, as an example, insulin being incorrectly delegated by a RN(registered nurse)	23
N	Lee 2015	Australia	Community home care	Assess the WISE Medicines Care model and explore nurses’, community care aides, patients and carers’ experiences and satisfaction with expanded roles Mixed Methods. Prospective before-after evaluation	467 patients received medicines support prior to implementation 572 patients’ post-implementation. 22 Registered nurses, 5 Enrolled nurses, 7 Community care aides and 18 patients and 10 carers participated in at least one focus group/interview. 6 Registered nurses, 2 Enrolled nurses, 3 Community care aides participated in both pre- and post-implementation interviews	Did not provide details of the types of medicines tasks delegated. Described that delegation of medicines support included prompting clients to self-administer medicines, removing medicines from packaging, crushing tablets, and assisting with administration of oral and topical medicine (no types mentioned). Additional tasks were also performed as well as medicine support	37
O	Owen 2009	UK	Community home care	Describe the implementation of insulin delegation Mixed Methods in-house service evaluation	12 healthcare assistants, 29 patients	Insulin	11
P	Randolph 2010	USA	Nursing home	Development and conducting a pilot program to determine the impact on patient health and safety of licensed nurses delegating medication administration to trained certified nursing assistants Mixed Methods service evaluation	22 Nurses	Did not provide details of the names of medicines delegated. Described that delegation of regularly scheduled medications including controlled substances, by the following routes: oral, topical, nasal, otic, optic, and rectal As-needed medications for bowel care or over-the-counter analgesic agents	28
Q	Reinhard 2006	USA	Assisted living facilities	To provide a national perspective on medication delivery in assisted living from the perspectives of state Board of Nursing executives. Qualitative interviews	42 State Board of Nursing executives	Did not provide details of the types of medicines delegated. Described that delegation of medicines occurs in conjunction with other tasks	27
R	Spellbring 2003	USA	Group Senior Assisted Housing	Describe the expansion of unlicensed caregivers’ function in Group Senior Assisted Housing Mixed Methods in-house service evaluation	97 patients, 176 Unlicensed staff	Medication includes oral, eye drops, inhalers, patches, and other topical preparations Injectable medications not permitted	20
S	(Young et al., 2016)	USA	Community home care	Evaluation of nurse delegation pilot project Mixed Methods in-house service evaluation	176 Registered nurses, 49 Home health aides, 54 administrators, 44 consumers	Did not provide details of the types of medicines delegated. Described that delegation of medicines occurs in conjunction with other tasks	39
T	(Young et al., 2008)	USA	Assisted living facilities	Describe the types and potential clinical significance of medication administration errors in assisted living Quantitative cross-sectional observational study	9 unlicensed assistive personnel, 510 patients	Oregon – oral and topical medication. The registered nurse can delegate insulin administration Washington - registered nurse delegate oral and topical medication, and non-registered support workers are not allowed to administer insulin New Jersey - medication aides are certified and complete state-required training, and registered nurses delegate administration of all medications	33

### 2.4 Data extraction and analysis

Data extraction was conducted by two authors (CS, KS) using a bespoke data extraction excel file and included details about the study participants, settings, study methods (Table 2) and outcomes significant to the review aims and objectives. Each paper was given a letter to act as identifier (Table 2).

**Fig. 2:**
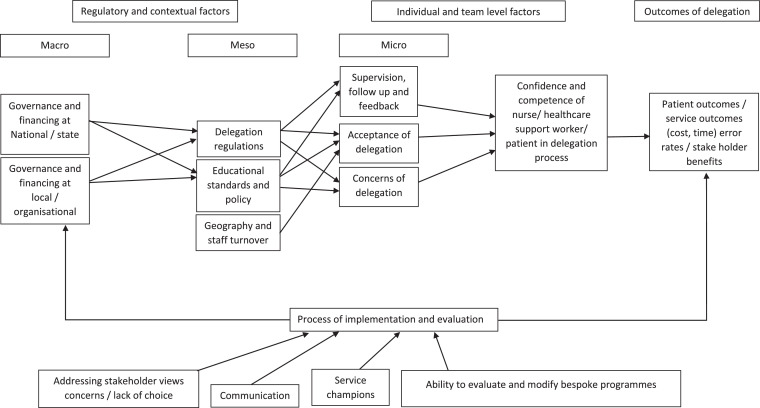
Delegation of medication administration Framework. Factors to consider in understanding delegation of medication administration.

The aim of the analysis was to synthesise evidence and develop a coherent theoretical framework including a network of constructs and the relationships between them (Dixon-wood et al., 2006). This was achieved by initial detailed analysis of the included studies to identify recurring themes which were then grouped into descriptive themes and sub-themes, by two authors (CS, KS). Critical Interpretative Synthesis requires constant reflexivity and ongoing critical orientation (Dixon-wood et al., 2006). Following initial analysis, authors (CS, KS) returned to manuscripts to confirm findings and extract further data relevant to emerging themes. This reflective and recursive process occurred until no further themes were emerging, and all appropriate data was extracted. Findings were then discussed and agreed with co-authors (FM, JM, KW, AC). Emerging findings were grouped as: a) macro level, pertaining to national and governmental influence; b) *meso* level, defined as organisational influence (e.g., a nursing home); and c) micro level, defined as individual level factors. To further understand the key factors that affect the delegation of medication administration, data were brought together to construct the ‘Delegation of medication administration Framework’ (Fig. 2). The framework provides a visual representation of the factors identified from the synthesis. The framework is not designed as a prescriptive formula, rather an aide to visually demonstrate the complex interaction of factors.

## 3 Results

Twenty of the 1556 identified articles (Fig. 1) met the inclusion criteria (Table 1).

### 3.1 Study characteristics and quality assessment

Table 2 describes the characteristics and quality assessment scores of included articles. Studies were undertaken in five countries: eight in the USA (B; G; L; P; Q; R; S; T), six in Sweden (A; C; E; I; J; M), four in the UK (D; H; K; O), one in Belgium (F) and one in Australia (N).

Five studies used qualitative methods (C; E; I; J; Q), six quantitative (A; B; F; G; L; T) and nine employed mixed methods (N; D; H; K; M; O; P; R; S). Seven studies describe and evaluate the introduction of new medication administration delegation programmes which were implemented into practice (N; D; H; K; O; P; R). The remainder provide insight into understanding current practice (C; E; I; J; Q; A; B; F; G; L; T; M; S).

All studies included results from community settings, including: assisted living facilities (Q; R; T), nursing homes (G; L; P) residential care (I), one reported data across multiple settings (B), and the remainder were in community homecare. Studies participants included nurses (C; E; F; G; H; I; M; N; P; S), non-registered support workers (A; B; D; G; H; F; O; K; J; R; T; N); management staff (F; G; L; Q; S); and patients (H; N; O; R; T). Dependant on country and setting, different titles were used and are described in detail in Table 2. Sample sizes within the studies ranged from 6 (K) to 6344 (L) (Table 2).

The methodological quality of included studies was average (mean Quality Assessment Tool for Studies with Diverse Designs score 25 (7–39) out of 48), mainly due to six low scoring studies, mostly service evaluations (D; H; K; M; O; R). Common methodological weaknesses were: lack of statistical assessment of reliability and validity of measurement tool(s); lack of or limited evidence of user involvement or of an explicit theoretical framework. Methodological strengths were: clear description of research setting; statement of aims/objectives in main body of report; and fit between research question and method (see appendix 2 and 3).

### 3.2 Delegation of medication administration

Five studies focused on a single type of medication administration delegated, of which insulin administration was delegated in four studies (D; H; K; O) and antiosteoporosis medication in one (L). Seven studies detailed a range of medication tasks and types of delivery eligible for delegation, including: topical medicines, controlled medicines, injectables (A; B; G; N; P; R; T). The remainder did not provide details of the type of medication delegated but indicated that medication administration was delegated alongside other tasks (Table 2).

### 3.3 Theoretical analysis

Delegation was found to be a complex phenomenon that is influenced by a myriad of interdependent factors at play at the macro, *meso*, micro level (see Fig. 2). Central to the interpretation of success of delegation was the relationship between the delegator and delegatee through which competence and confidence in delegation is acquired. This relationship was influenced by complex array of personal, educational and systems factors, the impact of which varied according to setting and context.

Findings are reported under four main themes:

1. Regulatory and contextual factors;
2. Individual and team level factors;
3. Outcomes of delegation; and
4. Process of implementation and evaluation.

Table 3 provides an overview of the main themes, associated sub themes and summative findings. Factors presented within themes can be viewed either as barriers or facilitators to delegation, depending on the context they are viewed within. For example, poor communication was a barrier, while appropriate communication of relevant information facilitated delegation. It is acknowledged that the themes influence and, in some cases, overlap with each other. Therefore, to avoid duplication of findings, data is presented within themes deemed most appropriate, yet acknowledging their presence and influence elsewhere.

**Table 3:** Major themes and sub-themes from the included studies, with summative finding.

Major themes	Sub themes	Summative finding
Regulatory and contextual factors		
	Governance and financing at the national, state, and organisational level	Legislation and policy was found to facilitate or impede delegation of medication administration. A lack of clear governance and communication of policy lead to confusion over what medication related tasks could be delegated and who held responsibility for delegating. Monitoring procedures and adherence to policy also varied. Fewer problems were reported where clear policies, governance and monitoring procedures were in place
	Educational standards and policy	Standards varied for the level of training required by non-registered support workers, including how training was designed, delivered, and assessed. The adequacy of training for medicines administration was a concern for many nurses, non-registered support workers and senior stakeholders
	Geography and staff turnover	Managing delegation over large geographical areas and without adequate staffing levels were reported as barriers at an organisational level

Individual and team level factors		
	Supervision, follow up and feedback	Delegation is a complex phenomenon, central to which is the relationship at the micro level between the nurse, non-registered support workers and patient. Adequate supervision, including assessment and monitoring of competencies, is key to developing confidence in the delegation process
	Acceptance of delegation	non-registered support workers, nurses, service managers and patients were largely accepting of delegation
	Concerns about delegation	Despite general acceptance, there were numerous concerns about delegation, including, coercion, safety, cost cutting and job erosion

Outcomes of delegation		
	Service efficiency	Delegation of medication administration was widely reported by nurses, non-registered support workers and service managers, to improve service efficiency and free time for registered nurse to focus on more complex nursing care or assessments of patients
	Improved patient care and error rates	Mixed findings are in contrast with anecdotal reports of positive impact of delegation on patient safety and error rates across a range of medication. No studies report on change in medication error rate directly attributable to non-registered support workers undertaking delegated medication administration
	Staff cohesion	The delegation process enhanced trust and helps to develop supportive and collaborative relationships between staff

Process of implementation and evaluation		
	Communication	Clear communication at all levels facilitated the delegation process, including communication of legislative and regulatory issues at national or state level; existence and communication of local delegation policy and procedures; communication within teams
	Stakeholder engagement	Evaluations of bespoke delegation programmes reported that the success of delegation hinged on stakeholder engagement from the start of implementation and strong leadership
	Service champions	Greater acceptance of delegation was reported to be influenced by the presence of a senior nurse or non-registered support worker ‘champions’, often those were already well known to staff
	Ability to evaluate and modify	Positively evaluated delegation programmes emphasised the importance of dedicating time for service evaluation, reflection, and flexibility to modify policy and practice

### 3.4 Theme 1. Regulatory and contextual factors

A range of regulatory and contextual factors acted as barriers or facilitators to implementing delegation of medication administration, including governance and financing at the national, state, and organisational level; educational standards and policy; and geography and staff turnover.

#### 3.4.1 Governance and financing at the national, state, and organisational level

At the macro level, Government and state level legislation and policy was found to facilitate or impede delegation of medication administration to non-registered support workers at the organisational level, depending on its consistency and clarity (L; Q). Lack of clarity led to confusion over what medication related tasks could be delegated and who held responsibility for delegating (B; M; N; Q).

A state level analysis of delegation regulation across the USA (Q) identified variation and lack of clarity over what tasks could be delegated in assisted living facilities; 24 states did not permit the delegation of medication administration, whereas in the 22 that did, regulations differed on the type of medication that non-registered support workers could administer. Interviews with Board of Nursing executives identified poor familiarity with delegation policy and a lack of consistency in the delivery, educational support, accountability, and monitoring of medication administration (Q). Differing interpretations of regulations were also reported, for example registered nurses in 10 states in the USA were held accountable for the process of delegation although this was not specified by law (Q). Elsewhere, concerns were raised from registered nurses and non-registered support workers about lack of clarity and fit between national level policy, the reality of delegation within local organisations and support to understand the legalities concerning responsibility for delegation (I; E). A separation of governing authorities for registered nurses and non-registered support workers in Sweden was reported to exacerbate difficulties in establishing lines of responsibility, working across different locations, maintaining team cohesion and provision of care (E). A need to provide training for non-registered support workers and registered nurses on the legalities of what can be delegated was suggested to improve poor levels of understanding (F; B, C; T).

Procedures to monitor and review delegation arrangements (e.g., on an annual basis) were in place in a number of studies (D; O; C; E; I; J), however, one study (J) reported that reviews were not always followed through. Three studies reported a lack of effective governance and monitoring procedures (I; J; Q). Others reported that regulations were not always adhered to, for example, a survey of medication aides across states in the USA found that some non-registered support workers were administering medication in ways that contravened their state regulations, including high risk medications such as insulin (B). As motivation for delegation was often driven by increased demand or lack of registered nurses’ capacity especially at evenings and weekends (M), delegation had become essential to service functioning (C; E; I; J; D). Pressure to meet demand was the reason given by some registered nurses who reported to delegate outside of regulations (C). Similarly, due to work pressures, some non-registered support workers were delegated medication administration without having their competencies assessed (A). Notably, fewer problems were reported by smaller scale delegation programmes where clear policies and governance procedures for delegation were in place and where staff engagement had taken place to address concerns and expectations around delegation (D; O; R).

#### 3.4.2 Educational standards and policy

Standards varied for the level of training required by non-registered support workers, including how training was designed, delivered, and assessed. In three studies (D; O; K), development of training was informed by prior assessment to establish the training needs and support required by non-registered support workers, or assessment of risk. Nine studies reported how trusts required non-registered support workers to undergo training specific to medication administration tasks (B; D; H; K; O**;** P; R; L; N). Of those studies, seven described how training was delivered: by registered nurses (R; P), independent educator and registered nurses (N), community practice educator (H), diabetes specialist nurses (D; O), and nurse consultant (K). Details of the length of training undertaken were provided by 7 studies (B; D; H: L; O; P; R) and ranged from 1 (O) to 13 days (P).

The adequacy of training was a concern for many registered nurses, non-registered support workers and senior stakeholders, particularly training and skills in relation to delegation of complex medication tasks (C; O; Q) and related indications and effects of medication (I). Several studies report bespoke training programmes, including four UK based studies on insulin delegation in community services (O; H; D; K; R). These programmes were developed with the input of senior staff, educational and clinical specialists. In bespoke projects, non-registered support workers and registered nurses described the need for training and continued professional development updates (H; O; R), suggesting participants were serious about their new responsibilities and aware of the complexity of medication administration (R).

In the USA, educational requirements differed according to setting and location (B; Q). A survey of medical aids in the USA found those working in nursing homes received more direct education on medication and direct supervision than those working in other community settings (B). Standards in assisted living facilities in the USA varied by location; in some states, registered nurses determined the degree of training required by non-registered support workers (Q).

Nine studies reported the development of local policy and governance for the delegation of medication administration, including standards for assessing, monitoring, and reviewing non-registered support workers competency (D; H; K; O**;** P; R, N; G; S). In Sweden, problems were reported in relation to registered nurses access to information about non-registered support workers level of training or capabilities to assist them in assessing competence for delegation (I; C). Improvements in knowledge and competences were reported following bespoke structured training programmes for non-registered support workers on administering specific medications (R; N; D; O). Finally, three articles described the provision of refresher training to non-registered support workers on medicines management associated with the delegated medication (N; D; P).

#### 3.4.3 Geography and staff turnover

Barriers to delegation were reported where too few non-registered support workers were employed within a local area, or where geographical distance impeded adequate supervision and monitoring (N; J). In contrast, delegation of insulin by non-registered support workers was reported to benefit patient care and team working across large rural areas (D). High non-registered support workers staff turnover made training and follow-up impractical (E; P; T).

### 3.5 Theme 2. Individual and team level factors

At the micro level, various factors were reported to influence acceptance and the development of competence and confidence in the delegation process. These included supervision, follow-up and feedback, acceptance of delegation and concerns about delegation.

#### 3.5.1 Supervision, follow up and feedback

As part of an effective working relationship, regular supervision and feedback on task activity was reported to be crucial for non-registered support workers ability to develop skills and gain confidence (N; S). There was consensus from registered nurses and non-registered support workers that adequate time was necessary to enable registered nurses to check non-registered support workers competencies against tasks and for both parties to develop confidence in the capability of individual non-registered support workers (F; C; M; I; N). Supervision also provided an opportunity for registered nurses to highlight future training needs of non-registered support workers (H). However, multiple studies reported that supervision did not always occur as expected (J; F; B; N; L). Findings from the USA indicated that adequate time and resources to support supervision was dependent on local regulations and that the extent of supervision was often determined by the state agency that licensed the assisted living facility (Q). Supervision was sometimes restricted by the care profile of patients, for example, non-registered support workers caring for more independent patients received less supervision than those caring for patient's dependant on care (F).

#### 3.5.2 Acceptance of delegation

In general, delegation was considered acceptable (N; O; H; P; F; R; S; Q; C; I) by non-registered support workers and registered nurses. Non-registered support workers were generally receptive to taking on new tasks and learning new skills (N), particularly if it would benefit patient care (O), help ease pressures on the wider team (H), or free registered nurses time (P). Registered nurses welcomed the inclusion of non-registered support workers within teams (N; S; F), valued the additional assistance and relief of workload pressure on qualified staff (H; R). Service managers were accepting of delegation in principle (F; S), but managers and registered nurses were less positive than non-registered support workers and consumers when asked about their confidence in implementing care, via a readiness to implement scale (S).

Four studies, which were bespoke programmes with inbuilt evaluation, assessed patients’ views on delegation. Predominately, patients were happy with the care provided by non-registered support workers in the delegation process, rating care as excellent or good (H; O; S; N). Patients noted that non-registered support workers were well trained (O) and would happily recommend the service to other patients (H), although not all patients were able to identify the type of worker providing care (N). Patients appreciated that delegation could benefit the service, for example, allowing registered nurses more time to treat patients with complex needs (N).

#### 3.5.3 Concerns about delegation

Delegation regulations usually stipulate that registered nurses and non-registered support workers have a choice if they want to delegate a task or take on a delegated task. However, findings from three studies suggest covert coercion to delegate, or accept delegated tasks, in order to retain employment or to ensure continuation of the service (A; I; E). For these reasons, registered nurses and non-registered support workers in some studies requested limiting delegation to simple, regularly repeated tasks considered safer for non-registered support workers to manage, such as oral administration of routine medications (F). Whereas tasks requiring greater degrees of judgment (such as performing injections, administration of controlled drugs, or multiple different medications) were considered less safe to delegate without adequate training (G; N). Five studies reported that safety concerns dissipated once registered nurses had observed delegation in practice (Q; C; O; I; N).

Delegation and its associated processes of training, assessment, supervision, and follow-up were reported to be time-consuming (F). Many registered nurses feared that delegation of medication administration was a cost-cutting exercise to reduce nurse staffing levels, (M; D; I), and threatened erosion of their job roles (D), particularly where non-registered support workers had additional responsibility due to reduced nurse staffing over evenings and weekends (M). Fearing liability, which could negatively impact on nurse registration and livelihood, some registered nurses avoided delegating medication administration, despite being time-consuming to perform themselves (I). non-registered support workers also expressed unease at being allocated tasks that were traditionally the remit of nurses, reporting tensions over potential nurse job erosion and devaluation (F).

### 3.6 Theme 3. Outcomes of delegation

Benefits cited from delegating medication administration from registered nurses to non-registered support workers were grouped as, service efficiency (inclusive of service costs and reduced work pressure on nurses); improved patient care; and staff cohesion.

#### 3.6.1 Service efficiency

Increased visits by non-registered support workers resulted in fewer nurse visits for patients requiring medication support for low complexity long-term conditions (N). Calculations by one study (H) found 2 non-registered support workers performed 47 visits to administer insulin to diabetic patients (*n* = 4) over a 12-week period; amounting to 80 h release time of registered nurses who would have undertaken the visits. A cost saving of £7.03 per hour is calculated by one study (D) for non-registered support workers to administer insulin in place of registered nurse. Reduced duplication of visits to the same patient by registered nurses was also reported (N; E). While staff widely reported that delegation improved service efficiency and enabled registered nurses to focus on more complex nursing care (F; I; P; D; H; N; O), additional costs need to be considered in association with increased nurse time for follow-up, supervision, training (F; I) and backfill of registered nurses performing those tasks (H).

#### 3.6.2 Improved patient care and error rates

Registered nurses considered that non-registered support workers involvement improved care planning for patients (O), resulted in early detection of problems (R), and increased continuity of the health-care delivery (C; D; O). Registered nurses were confident that non-registered support workers would contact them if they had concerns with an unfamiliar task (E; R) and patient safety was not considered to be affected (R; C; O; H). Non-registered support workers were thought to facilitate more timely administration of medication (C), particularly insulins (D; O), increased patient stability (Q) and promoted patient health and independence (S). No studies measured these outcomes.

Evidence of impact on medication errors was the subject of four studies (M; T; P; L). One study (M) reported that 31 of 68 medication errors by registered nurse in a four-year period were errors in medication administration delegated to non-registered support workers, including: wrong medicine; wrong patient; failure to administer; and wrong information passed from nurses. One study (T) reported similar types of errors: observations of 29 non-registered support workers across 12 assisted living settings, giving medications to 510 residents revealed 1373 errors (*N* = 4866 total observations, average error rate of 30% (range 13–40%). Four adverse drug events were rated as having potential for clinical harm (2 wrong dose and 2 unauthorised drugs). 70% (961/1373) of errors were related to medication administered at the wrong time; the remaining was accounted for by wrong dose (13%, 179/1373); omitted dose (11%, 151/1373); extra dose (4%, 55/1373); unauthorised drug (2%, 27/1373); and wrong drug (>1%) (T). One study (P) reported that the introduction of non-registered support workers with the title of Medication Technicians into six nursing homes in Arizona state resulted in a mean reduction of medication error rates of 10.4% (Licensed practice nurse = 10.12%, registered nurse  = 11.54%) vs 6.6% (Licensed practice nurse = 7.25%, registered nurse = 2.75%, non-registered support workers= 6.06%). In contrast, a larger study of nursing homes across 23 states in the USA reported that those nursing homes employing non-registered support workers as Medication Technicians were more likely to have medication error rates (10% vs 7%) than those homes who did not (L).

#### 3.6.3 Staff cohesion

Where delegation was sufficiently supported in practice, both registered nurses and non-registered support workers noted improvements in team cohesion, trust, non-registered support worker confidence, motivation, and morale (N; H; O; F; Q; J). Non-registered support workers felt more respected, valued, and supported by nurse colleagues (H; O; J; N; P). Enhanced team working facilitated monitoring of medications more closely (Q), information exchange that benefited both parties (J), and improved the comfort and care of dependent patients (F).

### 3.7 Theme 4. Process of implementation

Several important facilitators/barriers to the process of implementing a delegation programme were identified. The findings suggest that if regulatory and contextual factors are favourable, and there is support towards developing delegation skills and knowledge, there is increased confidence in the delegation process. The following subthemes ran across all themes: communication, stakeholder engagement, service champions, and ability to evaluate and modify service.

#### 3.7.1 Communication

At a local level, involvement of non-registered support workers within team meetings helped prevent misunderstanding (A; B; J) and was regarded as an important tool in creating a trusting atmosphere (E). Investing time for effective communication between non-registered support workers and registered nurses was a key recommendation for improved service (J). Barriers to safe delegation included poor communication of changes to branding and appearance of medications (J).

#### 3.7.2 Stakeholder engagement

Early stakeholder engagement with staff from multiple clinical and administrative roles (consultants, general practitioners, specialist nurses, registered nurses, non-registered support workers and managers) to involve staff and address concerns about delegation appeared to increase understanding and reduce implementation barriers (D; H; O; S). Stakeholder engagement was also reported to help address issues of accountability, supervision, patient safety and ratification of the service policy (K; O; R; D; N). Three studies reported that multi-professional involvement in the development of delegation policy, training and assessment process was essential. However, it required a lot of organising and time investment to gain buy-in from staff (H; O: N).

#### 3.7.3 Service champions

Strong leadership from senior staff and service champions, who were well known and approachable, helped influence acceptance and managed expectations around delegation (R; N; J).

#### 3.7.4 Ability to evaluate and modify

Delegation programmes that were positively evaluated emphasised the importance of dedicated time for service evaluation, allowing time for reflection on progress and the flexibility to modify policy and practice (R; O), or the length of required training (D).

## 4 Discussion

This is the first systematic review of the delegation of medication administration from registered nurses to non-registered support workers in community settings. The methodological quality of the included studies is of average standard. While the included studies do provide beneficial insights into implementation and current practice, there is a lack of robust pre-and-post comparative testing around many facets, including cost, clinical outcomes, and medication errors. Furthermore, there are noticeable gaps in the evidence base, for example, patient outcomes or experience. Subsequently, this limits the capacity to judge if medicines delegation is appropriate in all cases. The review found that delegation of medication administration occurs, and has been the subject of research, in several high-income countries and across a range of community care settings. Job titles for non-registered support workers are diverse (Blay and Roche, 2020). Some non-registered support workers have a dedicated role in medication administration (e.g., medication aides in USA) and others undertake medication administration as part of a wider role. Delegation itself was found to be a complex phenomenon influenced by multiple, and in some cases interlinked factors at the macro, *meso*, micro level.

### 4.1 Macro to micro level influence

As set out in Fig. 2, the extent to which medication administration was formally delegated was influenced by national/state level (macro) and organisational (*meso*) level regulation. Variation was found in governance and educational standards for delegation. Where lack of clarity was reported at the macro level, this filtered through to *meso* and micro levels, resulting in confusion about what medication administration could be delegated and who held responsibility (Budden, 2012; Kapborg and Svensson, 1999; Lee et al., 2015; Reinhard et al., 2006). Lack of fit between policy and local work arrangements led to delegation occurring outside of regulation, incurring potential risk to patient safety (Bystedt et al., 2011). In these situations, acceptance of medication administration delegation was poor, viewed as a burden, or risk to patient care (Gransjon-Craftman et al., 2016). In contrast, findings indicate that these barriers are overcome where clear and consistent policy exists, backed by training on medication administration and processes for regulation and review (Cook, 2015; Owen 2009; Spellbring and Ryan, 2003). Under these conditions, staff acceptance was high, and benefits were identified for patient care and team members (Cook, 2015; Owen 2009; Spellbring and Ryan, 2003). However, many studies were small scale evaluations and there is a need for more robust, larger scale independent investigation to verify such findings.

At the micro level, central to a successful delegation process was the supervisory relationship between the delegator and delegatee, whereby mutual respect and confidence in non-registered support worker competence was nurtured. Confidence and acceptance of delegation was to some extent dependent on adequate supervision contact, clear role boundaries, robust training and clear procedures governing delegation of medicines administration (Owen, 2009; Dutton et al., 2018; Cook, 2015; Gregory, 2019; Spellbring and Ryan, 2003). Where there was felt to be inadequate time for the registered nurse and non-registered support workers to get to know each other, assess competency or to follow-up on delegated tasks, both registered nurses and non-registered support workers were less accepting of delegation (De Vliegher et al., 2016; Bystedt et al., 2011; Kapborg and Svensson, 1999; Gransjon-Craftman et al., 2016; Lee et al., 2015). These findings align with those reported in the wider literature on delegation, for example Perry et al. (2003), found delegation inhibited by a poor distinction between registered nurses add non-registered support workers roles and activities undertaken in nursing homes. In acute settings, good communication, strong relationships, positive attitudes and the level of non-registered support worker competence and knowledge were identified as key to successful delegation (Gravlin and Bittner 2020; Campbell et al., 2020). Direct supervision has been shown to be effective at producing change, assessing clinical performance, and encouraging interaction between the supervisor and supervisee (Snowdon et al., 2017). The ability to communicate well, monitor and provide feedback has been shown to help develop mutual respect between nurse and non-registered support workers in delegation practice in other settings (Gillen and Graffin 2010; Anthony and Vidal, 2010; Munn et al., 2013). Furthermore, interventions to improve teamwork, communication and delegation between registered nurse and non-registered support workers in acute settings can impact on patient satisfaction and outcomes (Campbell et al., 2020).

### 4.2 Outcomes of delegation

Evidence of impact is mainly self-report, with no patient outcomes reported other than satisfaction with the delegation process, which in itself was limited and from pilot studies. If healthcare is to be patient centred, a lack of reporting on patient experiences reduces insights into how appropriate delegation is in all cases. It can be argued that successful delegation is where care provided by the non-registered support worker is equivalent to that of the registered nurses, therefore delegation may not improve care but may prevent deterioration of care, or missed care, that can occur due to a lack of resources (Bittner and Gravlin, 2009; Kalisch, 2006). However, a wide range of potential benefits are reported that require further investigation to truly have a rounded idea of how delegation is influencing practice, including: timeliness of medication administration; early detection of problems; improved consistency of care; rapport with patients; teamwork and job satisfaction for nurse and non-registered support workers. The theoretical model (Fig. 2) hypothesises that outcomes will be determined by the relationship between delegator and delegatee, which is influenced by a range of *meso* and macro level factors affecting understanding and acceptance of the delegation process. Further research is recommended to develop and test this model.

### 4.3 Patient safety

The consensus from stakeholders (managers and registered nurses), was that delegation, particularly for higher risk medicines (e.g., insulin), requires tailored training and a high level of regulation, monitoring, and review. Findings indicate that patient safety may be at risk where governance and regulation is poor and concerns were raised by stakeholders over non-registered support workers skill level (Bystedt et al., 2011; Owen, 2009); adequacy of training around complex medication tasks (Reinhard et al., 2006), and on indications and effects of medication (Gransjon-Craftman et al., 2016). As the delegation of medicines administration is primarily driven by increasing demand for services, the impact on patient safety requires careful monitoring and robust reporting for evaluation. There were concerns that task focused delegation may result in missed opportunities to provide holistic care or prevent detection of change in a patients’ condition. In community settings, the traditional registered nurse role is known to be multifaceted, requiring clinical knowledge and skills in decision making, risk assessment, palliative care, and health promotion (Heath, 2012). This holistic approach at the heart of nursing may be at threat from a rationing or task-orientated approach which has been associated with increased incidents of missed care, lower satisfaction, and poor staff retention (Mandal et al., 2020). The evidence reported in this review from four studies (Kapborg and Svensson, 1999; Young et al., 2008; Randolph and Scott-Cawiezell, 2010; Hughes et al., 2006) presents mixed findings on medication errors related to non-registered support workers and a lack of comparison of error rates between registered nurses and non-registered support workers. Findings reiterate the importance of creating a patient safety culture that encourages reporting and learning from errors as this has been linked to improved patient outcomes (Bonner et al., 2009, Thomas et al., 20122). Level of education and knowledge of clinical guidelines have also been associated with improved patient safety culture in nursing homes (Titlestad et al., 2018), emphasising the complex interplay between these factors.

### 4.4 Implementation

Findings identified several factors that influence the implementation process of delegation, including good communication, stakeholder engagement and use of service champions (Cook, 2015; Owen 2009; Spellbring and Ryan, 2003). Practicalities of geography and employment were examples of contextual factors that influenced implementation of delegation. The importance of building in a process to evaluate and refine delegation initiatives was also stressed. However, implementation was not the primary focus of studies and further research guided by models of implementation would enhance understanding of facilitators to implementation, potential scalability, and sustainability of delegation initiatives. The Consolidated Framework for Implementation Research could be considered (Damschroder et al., 2009), although it is important to find the right framework to suit the healthcare innovation being implemented (Moullin et al., 2015). A comprehensive economic evaluation is also recommended that takes into consideration the costs of training, supervision and governance issues relating to the delegation of medication administration. Increasing the evidence base will provide greater understanding of the initial and long-term sustainability of medicine delegation, which is currently lacking.

### 4.5 Strengths and limitations

The flexibility of the Critical Interpretative Synthesis approach enabled identification of relevant evidence on the delegation of medication administration from studies with varied aims and designs, which enhanced the depth of the review findings and facilitated the development of a theoretical model of this practice. The limits of Critical Interpretative Synthesis in terms of transparency of process and trustworthiness were countered in this review by providing a detailed search strategy and record of process. Identified studies were all from high income countries, potentially limiting transferability of the results across all clinical and professional specialties internationally. Gaps in the reported evidence base and a lack of robust pre-and-post comparative testing reduces the capacity to judge if delegation of medication administration is appropriate in all case. However, included studies cover a wide range of facilities and countries, providing a rich representation of delegation of medication administration from registered nurse to non-registered support workers in community settings. Gaining insight into factors that influence implantation are a further strength of the review, although these should be treated with caution as evaluation of implementation was not the main focus of the included studies.

## 5 Conclusion

In conclusion, delegation is a complex process influenced by many interrelating factors, central to which is the relationship between the delegator and the delegatee. Due to the increased risk associated with the complexity of medication administration, clear and consistent regulatory and governance frameworks and procedures are crucial. Findings suggest that the quality, clarity, and consistency of governance measures to support delegation may influence outcomes, however more research is needed to measure outcomes for patients, staff, and services, including error rates. Certainly, delegation is more acceptable within a framework that adequately supports the process, backed by appropriate policy, skills, training and adequately resourced supervisory arrangements. As delegation is already occurring in community settings in many countries, it is important to understand how to best ensure that it develops as an effective service in a way that puts patient safety first. Findings highlight key barriers and facilitators to be taken into consideration by healthcare services implementing delegation of medication administration. This review calls for robust pre-and-post comparative testing around all facets of clinical outcomes and medication errors associated with delegation of medication administration.

## Declaration of Competing Interest

The authors declare no conflict of interest.

## Appendix Supplementary materials

Image, application 1
