# Supplementary material for: Delegation of medication administration from registered nurses to non-registered support workers in community care settings: A systematic review with critical interpretive synthesis
Source: Int J Nurs Stud. 2022 Feb;126:104121. doi: 10.1016/j.ijnurstu.2021.104121 (PMC8803545; doi:10.1016/j.ijnurstu.2021.104121)
Supplement: Supplementary file 1 [file mmc1.pdf]

Appendix A. Search String used within MEDLINE database

#	Query	Results
1	(MH "Nurses, Community Health")	847
2	(MH "Nurses")	39,099
3	(MH "Community Health Nursing")	19,539
4	AB (unregistered N3 assistant* OR unlicensed N3 assistant*) OR TI (unregistered N3 assistant* OR unlicensed N3 assistant*)	23
5	(MH "Nursing Assistants")	4183
6	(MH "Physician Assistants")	5672
7	(MH "Allied Health Personnel")	11,741
8	AB ("healthcare support worker*" OR "health care support worker*") OR TI ("healthcare support worker*" OR "health care support worker*")	84
9	AB ("health care assistant*" OR "healthcare assistant*") OR TI ("health care assistant*" OR "healthcare assistant*")	798
10	AB ("ass* practitioner*") OR TI ("ass* practitioner*")	632
11	S1 OR S2 OR S3 OR S4 OR S5 OR S6 OR S7 OR S8 OR S9 OR S10	80,479
12	(MH "Community Health Services")	31,619
13	(MH "Home Health Nursing")	349
14	(MH "Home Care Services")	33,532
15	(MH "Home Nursing")	8539
16	(MH "General Practice")	13,319
17	(MH "General Practitioners")	7952
18	(MH "Family Practice")	65,019
19	(MH "Primary Health Care")	77,783
20	(MH "Ambulatory Care")	43,098
21	AB (community N5 health N5 team) OR TI (community N5 health N5 team)	1317
22	AB ("primary care") OR TI ("primary care")	113,983
23	AB "community provider" OR TI "community provider"	66
24	(MH "Home Care Agencies")	1338
25	(MH "Assisted Living Facilities")	1377
26	(MH "Residential Facilities")	5439
27	AB ("sheltered living") OR TI ("sheltered living")	38
28	AB ("nursing home") OR TI ("nursing home")	21,305
29	(S12 OR S13 OR S14 OR S15 OR S16 OR S17 OR S18 OR S19 OR S20 OR S21 OR S22 OR S23 OR S24 OR S25 OR S26 OR S27 OR S28)	344,561
30	(MH "Delegation, Professional")	570
31	(MH "Personnel Delegation")	308
32	(MH "Preceptorship")	5100
33	AB (task N5 delegat*) OR TI (task N5 delegat*)	619
34	AB (assig* N5 responsib*) OR TI (assig* N5 responsib*)	861
35	AB (auth* N5 responsib*) OR TI (auth* N5 responsib*)	2398
36	AB (auth* N5 task*) OR TI (auth* N5 task*)	1433
37	AB (hand over N5 task*) OR TI (hand over N5 task*)	54
38	AB (entrust* N5 responsib*) OR TI (entrust* N5 responsib*)	63
39	S30 OR S31 OR S32 OR S33 OR S34 OR S35 OR S36 OR S37 OR S38	11,249
40	(MH "Medication Errors")	13,211
41	(MH "Medication Systems")	876
42	(MH "Preventive Medicine")	11,712
43	AB ("medication admin*") OR TI ("medication admin*")	3055
44	AB ("pharmaceutical thera*") OR TI ("pharmaceutical thera*")	675
45	AB "drug admin*" OR TI "drug admin*"	58,960
46	S40 OR S41 OR S42 OR S43 OR S44 OR S45	86,862
47	S11 AND S29 AND S39	132
48	S11 AND S29 AND S39 AND S46	9
49	S11 AND S29	11,937
50	S11 AND S39	674
51	S11 AND S46	542
52	S29 AND S39	1235
53	S29 AND S46	2856
54	S39 AND S46	96
55	S11 AND S29 AND S39	132
56	S11 AND S39 AND S46	16
57	S11 AND S29 AND S46	93
58	S39 AND S29 AND S46	18
59	S50 OR S52 OR S55 OR S56 OR S57 OR S58	374

Limiters - English Language; Age Related: All Adult: 19+ years; Language: English. Expanders - Apply equivalent subjects. Search modes - Boolean/Phrase.

Appendix B. Quality Assessment Tool scoring guidance notes for Studies with Diverse Designs (Quality Assessment Tool For Studies With Diverse Designs)

Question	Criteria	0 = Not at all	1 = Very slightly	2 = Moderately	3 = Complete
**1**	**Explicit theoretical framework**	No mention at all.	Reference to broad theoretical basis.	Reference to a specific theoretical basis.	Explicit statement of theoretical framework and/or constructs applied to the research.
**2**	**Statement of aims/objectives in main body of report**	No mention at all.	General reference to aim/objective at some point in the report including abstract.	Reference to broad aims/objectives in main body of report.	Explicit statement of aims/objectives in main body of report.
**3**	**Clear description of research setting**	No mention at all.	General description of research area and background, e.g., 'in primary care'.	General description of research problem in the target population, e.g., 'among GPs in primary care'.	Specific description of the research problem and target population in the context of the study, e.g., nurses and doctors from GP practices in the east midlands.
**4**	**Evidence of sample size considered in terms of analysis**	No mention at all.	Basic explanation for choice of sample size. Evidence that size of the sample has been considered in study design.	Evidence of consideration of sample size in terms of saturation/information redundancy or to fit generic analytical requirements.	Explicit statement of data being gathered until information redundancy/saturation was reached or to fit exact calculations for analytical requirements.
**5**	**Representative sample of target group of a reasonable size**	No statement of target group.	Sample is limited but represents some of the target group or representative but very small.	Sample is somewhat diverse but not entirely representative, e.g., inclusive of all age groups, experience but only one workplace. Requires discussion of target population to determine what sample is required to be representative.	Sample includes individuals to represent a cross section of the target population, considering factors such as experience, age and workplace.
**6**	**Description of procedure for data collection**	No mention at all.	Very basic and brief outline of data collection procedure, e.g., 'using a questionnaire distributed to staff'.	States each stage of data collection procedure but with limited detail, or states some stages in details but omits others.	Detailed description of each stage of the data collection procedure, including when, where and how data were gathered.
**7**	**Rationale for choice of data collection tool(s)**	No mention at all.	Very limited explanation for choice of data collection tool(s).	Basic explanation of rationale for choice of data collection tool(s), e.g., based on use in a prior similar study.	Detailed explanation of rationale for choice of data collection tool(s), e.g., relevance to the study aims and assessments of tool quality either statistically, e.g., for reliability & validity, or relevant qualitative assessment.
**8**	**Detailed recruitment data**	No mention at all.	Minimal recruitment data, e.g., no. of questionnaire sent, and no. returned.	Some recruitment information but not complete account of the recruitment process, e.g., recruitment figures but no information on strategy used.	Complete data regarding no. approached, no. recruited attrition data where relevant, method of recruitment.
**9**	**Statistical assessment of reliability and validity of measurement tool(s) (Quantitative only)**	No mention at all.	Reliability and validity of measurement tool(s) discussed, but not statistically assessed.	Some attempt to assess reliability and validity of measurement tool(s) but insufficient, e.g., attempt to establish test–retest reliability is unsuccessful, but no action is taken.	Suitable and thorough statistical assessment of reliability and validity of measurement tool(s) with reference to the quality of evidence as a result of the measures used.
**10**	**Fit between stated research question and method of data collection (Quantitative)**	No research question stated.	Method of data collection can only address some aspects of the research question.	Method of data collection can address the research question but there is a more suitable alternative that could have been used or used in addition.	Method of data collection selected is the most suitable approach to attempt answer the research question
**11**	**Fit between stated research question and format and content of data collection tool e.g., interview schedule (Qualitative)**	No research question stated.	Structure and/or content only suitable to address the research question in some aspects or superficially.	Structure & content allows for data to be gathered broadly addressing the stated research question(s) but could benefit from greater detail.	Structure & content allows for detailed data to be gathered around all relevant issues required to address the stated research question(s).
**12**	**Fit between research question and method of analysis**	No mention at all.	Method of analysis can only address the research question basically or broadly.	Method of analysis can address the research question but there is a more suitable alternative that could have been used or used in addition to offer greater detail.	Method of analysis selected is the most suitable approach to attempt answer the research question in detail, e.g., for qualitative IPA preferable for experiences vs. content analysis to elicit frequency of occurrence of events, etc.
**13**	**Good justification for analytical method selected**	No mention at all.	Basic explanation for choice of analytical method	Fairly detailed explanation of choice of analytical method.	Detailed explanation for choice of analytical method based on nature of research question(s).
**14**	**Assessment of reliability of analytical process (Qualitative only)**	No mention at all.	More than one researcher involved in the analytical process but no further reliability assessment.	Limited attempt to assess reliability, e.g. reliance on one method.	Use of a range of methods to assess reliability, e.g. triangulation, multiple researchers, varying research backgrounds.
**15**	**Evidence of user involvement in design**	No mention at all.	Use of pilot study but no involvement in planning stages of study design.	Pilot study with feedback from users informing changes to the design.	Explicit consultation with steering group or statement or formal consultation with users in planning of study design.
**16**	**Strengths and limitations critically discussed**	No mention at all.	Very limited mention of strengths and limitations with omissions of many key issues.	Discussion of some of the key strengths and weaknesses of the study but not complete.	Discussion of strengths and limitations of all aspects of study including design, measures, procedure, sample & analysis.

Appendix C. Individual and averaged combined scores of included studies

Scorer	paper ID	Lead author and year	1	2	3	4	5	6	7	8	9	10	11	12	13	14	15	16	Individual score	Average combined score
KS	a	(Axelsson and	0	3	3	3	3	2	2	3	1	3	2	3	1	0	0	2	31	29
CS	a	Elmstahl, 2004)	0	3	3	3	3	2	1	3	0	2	2	2	0	0	0	2	26	
KS	b	Budden 2012	0	3	3	3	2	3	2	3	0	3	1	3	2	0	0	2	30	28
CS	b		0	2	2	3	3	2	1	3	0	3	1	2	2	0	0	1	25	
KS	c	Bystedt 2011	3	2	3	2	2	3	2	1	0	3	3	3	3	1	0	2	33	34
CS	c		3	3	3	2	3	3	3	3	0	0	3	3	2	0	0	3	34	
KS	d	Cook 2015	0	1	2	0	1	1	0	0	0	0	0	0	0	0	1	0	6	7
CS	d		0	1	3	0	1	1	0	1	0	0	0	1	0	0	0	0	8	
KS	e	Craftman et al. 2012	2	3	2	2	2	3	3	1	0	0	3	3	2	3	1	2	32	30
CS	e		2	3	2	1	2	2	1	2	0	0	3	3	2	1	2	2	28	
KS	f	De Vliegher 2016	1	3	3	1	3	3	2	1	0	0	3	3	2	3	0	2	30	28
CS	f		1	3	3	1	2	2	1	1	0	0	3	2	1	3	0	2	25	
KS	g	Dupler et al. 2015	1	3	3	3	2	2	3	2	1	3	0	3	3	0	1	1	31	29
CS	g		0	3	3	3	2	2	3	1	1	2	0	2	2	0	1	1	26	
KS	h	Dutton et al. 2018	0	1	2	0	1	1	0	1	0	0	0	0	0	0	0	0	6	7
CS	h		0	1	3	0	1	1	0	1	0	0	0	1	0	0	0	0	8	
KS	i	Granson - Craftman	2	3	3	2	2	3	3	1	0	0	3	3	3	3	0	2	33	31
CS	i	2016	2	2	3	2	1	2	2	2	0	0	3	3	2	2	0	2	28	
KS	j	Granson - Craftman	1	1	2	1	1	3	3	1	n/a	n/a	2	3	3	3	0	2	26	24
CS	j	2014	1	3	2	1	1	2	2	1	0	0	2	2	2	1	0	2	22	
KS	k	Gregory 2019	0	0	2	0	0	1	1	0	n/a	n/a	1	0	0	0	1	0	6	7
CS	k		0	1	3	0	1	1	0	1	0	0	0	1	0	0	0	0	8	
KS	l	Hughes 2006	0	3	3	2	3	3	3	3	3	3	0	3	3	0	0	2	34	32
CS	l		0	3	2	1	3	3	3	2	3	3	0	3	2	0	0	2	30	
KS	m	Kapborg 1999	1	2	3	1	2	3	1	2	0	2	3	2	1	1	0	1	25	23
CS	m		0	3	2	2	1	1	2	2	0	0	2	2	2	1	0	0	20	
KS	n	Lee 2015	1	3	3	2	2	3	2	3	2	3	2	3	2	2	3	3	39	37
CS	n		1	3	2	2	2	2	2	3	1	2	2	2	2	2	3	3	34	
KS	o	Owen 2009	0	1	2	0	2	1	0	0	0	0	1	1	0	0	2	1	11	11
CS	o		0	2	3	0	2	1	0	1	0	0	0	1	0	0	0	0	10	
KS	p	Randolph 2010	3	3	2	1	1	2	2	1	1	2	3	3	3	1	1	1	30	28
CS	p		3	3	2	1	1	1	2	1	1	1	2	2	2	1	1	1	25	
KS	q	Reinhard 2006	2	3	3	3	3	2	2	1	0	0	2	3	2	1	1	1	29	27
CS	q		2	2	3	3	2	2	1	1	0	0	2	2	1	2	0	1	24	
KS	r	Spellbring 2003	0	1	3	1	2	1	1	2	0	0	2	2	1	1	2	1	20	20
CS	r		0	1	3	2	2	2	0	2	0	0	2	2	0	0	1	2	19	
KS	s	(Young et al., 2016)	0	3	2	3	3	3	2	3	3	3	3	3	3	3	3	1	41	39
CS	s		0	2	3	2	3	3	3	2	3	3	2	3	2	2	3	1	37	
KS	t	(Young et al., 2008)	0	3	3	3	3	3	2	3	3	3	0	3	2	0	1	3	35	33
CS	t		0	3	3	2	2	3	2	2	2	2	0	2	2	0	2	3	30	
